# Supplementary material for: Introducing Semi-Interpenetrating Networks of Chitosan and Ammonium-Quaternary Polymers for the Effective Removal of Waterborne Pathogens from Wastewaters
Source: Polymers (Basel). 2023 Feb 22;15(5):1091. doi: 10.3390/polym15051091 (PMC10007103; doi:10.3390/polym15051091)
Supplement: Supplementary file 1 [file polymers-15-01091-s001.zip › polymers-2213081-supplementary.pdf]

Introducing semi-interpenetrating networks of chitosan and ammonium-quaternary polymers for the effective removal of waterborne pathogens from wastewaters

Iulia E. Neblea, Anita-Laura Chiriac, Anamaria Zaharia, Andrei Sarbu, Mircea Teodorescu, Andreea Miron, Lisa Paruch, Adam M. Paruch, Andreea G. Olaru, Tanta-Verona Iordache

Table S1. The main characteristic bands of semi-IPN hydrogels based on monomer VBTAC, crosslinker MBA and chitosan, and of the three types of chitosan (CC, CCH and SHC)

Sample	υ_{OH}	υ_{CH}, υ_{CH2}	$\upsilon_{C=O}$	υ_{-NH}	υ $_{N+(CH3)3}$
**CC**	3410	2886	1657	1562	-
**CCH**	3353	2869	1646	1568	-
**SHC**	3317	2891	1659	1566	-
**poly(VBTAC)**	3412	3028, 2928	1645	-	1482
**CC-IPN$_2$**	3412	3020, 2928	1653	1551	1480
**CC-IPN$_3$**	3407	3020, 2920	1660	1547	1484
**CC-IPN$_4$**	3445	3020, 2945	1654	1564	1489
**CCH-IPN$_2$**	3413	3024, 2924	1651	1552	1483
**CCH-IPN$_3$**	3407	3022, 2924	1655	1554	1483
**CCH-IPN$_4$**	3385	3023, 2921	1647	1563	1483
**SHC-IPN$_2$**	3427	3022, 2924	1655	1560	1487
**SHC-IPN$_3$**	3422	3028, 2922	1650	1560	1487
**SHC-IPN$_4$**	3407	3022, 2928	1628	1569	1482

Table S2. Specific decomposition temperatures and mass loss of the chitosan-based hydrogels and of the three types of chitosan (CC, CCH and SHC)

Sample	T_{d1},°C	T_{d2},°C	T_{d3}, °C	T_{d4}, °C	Mass loss, %
**CC-IPN$_2$**	87	265	317	429	77.98
**CC-IPN$_3$**	89	277	307	430	78.03
**CC-IPN$_4$**	92	272	300	409	76.85
**CC**	-	287	-	-	69.35
**CCH-IPN$_2$**	90	266	316	428	75.61
**CCH-IPN$_3$**	92	267	305	427	79.89
**CCH-IPN$_4$**	96	278	-	406	78.58
**CCH**	-	288	-	-	68.49
**SHC**	88	-	305	-	55.02
**SHC-IPN$_2$**	89	264	321	429	80.61
**SHC-IPN$_3$**	94	267	304	427	80.51
**SHC-IPN$_4$**	96	276 (hump)	301	408/426 (hump)	80.57
**polyVBTAC**	90	295	-	406	84.25
